# Credibility of Scientists: Barrow and Conrad Respond

**Published:** 2006-03

**Authors:** Craig S. Barrow, James W. Conrad

**Affiliations:** The Dow Chemical Company, Washington, DC, E-mail: cbarrow@dow.com; American Chemistry Council, E-mail: james_conrad@americanchemistry.com

We appreciate Goozner’s compliment that our commentary (Barrow and Conrad 2006) demonstrates “a sophisticated understanding of the nuances of the Federal Advisory Committee Act.” We wish we could take credit for “accurately point[ing] out that the act draws a distinction between conflicts of interest … and bias,” except that it does not—as we noted; federal rules under the Ethics in Government Act (1978) make the distinction. We did not, however, incorrectly misrepresent the Center for Science in the Public Interest’s (CSPI) basis for opposing the nominations of two scientists to sit on a U.S. Environmental Protection Agency (EPA) panel. We said that the CSPI opposed them because they were “funded by industry” (Barrow and Conrad 2006). Goozner characterizes this statement as implying that the scientists were only biased, whereas in his view the scientists really “were covered by the conflict of interest standard” because they “currently or previously worked for DuPont.” Alas, the scientists did not have a conflict of interest under the federal standard, which only applies to current employment or ownership (Office of Government Ethics 1997). The CSPI’s own press release makes clear that one of the two scientists, an academic, “four years ago conducted 3M’s $1.3-million study of … PFOA,” and that the other scientist, “[p]rior to working for [his current employer], spent many years working for DuPont ….” (CSPI 2004). Neither scientist worked for DuPont, or had a conflict of interest under federal rules, when he was being considered for the U.S. EPA panel.

In her letter, Sass cites four studies, involving three politically controversial chemicals, purporting to show that industry-funded research is more likely to find no adverse effects from the chemical studied, whereas government-funded studies are more likely to detect such effects. The authors of one of those studies at least recognized that these findings have two plausible interpretations: either “industry-funded scientists [are] under real or perceived pressure to find or publish only data suggesting negative outcomes,” or “government-funded scientists [are] under real or perceived pressure to find or publish only data suggesting adverse outcomes …” (vom Saal and Hughes 2005). Pielke (2005) observed that such obsessive focus on funding leads journalists in particular to conclude that “research findings are ‘in the eye of the beholder,’” a result he believes is “damaging to science and its role in policy.”

Sass urges the U.S. government and the National Academies to adopt more stringent conflict of interest guidelines, quoting a *Lancet* 2002 editorial that actually addressed manipulation of scientific panels by politicians. In an earlier commentary, more to the point, the editor of The *Lancet* (Horton 1997) argued that financial conflicts “may not be [more] influential” than biases and that “interpretations of scientific data will always be refracted through the experiences and biases of the authors.” He contended that disqualifying researchers from writing editorials or reviews because of their “associations” with industry “may harm free discussion in science.” Horton (1997) concluded that “[t]he only way to minimize bias among interpretations is to allow maximum dialogue from all parties, irrespective of their interests.” Making government conflict or bias rules more exclusionary will not serve that end.
